# Quality assurance and quality control reporting in untargeted metabolic phenotyping: mQACC recommendations for analytical quality management

**DOI:** 10.1007/s11306-022-01926-3

**Published:** 2022-08-27

**Authors:** Jennifer A. Kirwan, Helen Gika, Richard D. Beger, Dan Bearden, Warwick B. Dunn, Royston Goodacre, Georgios Theodoridis, Michael Witting, Li-Rong Yu, Ian D. Wilson, the metabolomics Quality Assurance and Quality Control Consortium (mQACC)

**Affiliations:** 1 Berlin Institute of Health at Charité – Universitätsmedizin Berlin, Metabolomics Platform, Anna-Louisa-Karsch-Str. 2, 10178 Berlin, Germany; 2 Max Delbrück Center, Robert-Rössle Strasse 10, 13125 Berlin, Germany; 3 School of Veterinary Medicine and Science, University of Nottingham, Sutton Bonnington Campus, Loughborough, LE12 5RD UK; 4 School of Medicine, Aristotle University of Thessaloniki, 54124 Thessaloníki, Greece; 5 BIOMIC_Auth, Center for Interdisciplinary Research and Innovation (CIRI-AUTH), 57001 Thermi, Greece; 6 Division of Systems Biology, U.S. Food and Drug Administration (FDA), National Center for Toxicological Research, Jefferson, AR 72079 USA; 7 Metabolomics Partners, 1065 Fronie Drive, Nesbit, MS 38651 USA; 8 Department of Biochemistry and Systems Biology, Institute of Systems, Molecular and Integrative Biology, University of Liverpool, BioSciences Building, Crown St., Liverpool, L69 7ZB UK; 9 Aristotle University of Thessaloniki, 54124 Thessaloníki, Greece; 10 Metabolomics and Proteomics Core, Helmholtz Zentrum München, Ingolstädter Landstraße 1, 85764 Neuherberg, Germany; 11 Division of Systems Medicine, Department of Metabolism, Digestion and Reproduction, Imperial College London, Hammersmith Campus, London, W12 0NN UK

**Keywords:** Quality assurance (QA), Quality control (QC), Reporting standards, Untargeted metabolomics, Mass spectrometry (MS), Nuclear magnetic resonance (NMR) spectroscopy

## Abstract

**Background:**

Demonstrating that the data produced in metabolic phenotyping investigations (metabolomics/metabonomics) is of good quality is increasingly seen as a key factor in gaining acceptance for the results of such studies. The use of established quality control (QC) protocols, including appropriate QC samples, is an important and evolving aspect of this process. However, inadequate or incorrect reporting of the QA/QC procedures followed in the study may lead to misinterpretation or overemphasis of the findings and prevent future metanalysis of the body of work.

**Objective:**

The aim of this guidance is to provide researchers with a framework that encourages them to describe quality assessment and quality control procedures and outcomes in mass spectrometry and nuclear magnetic resonance spectroscopy-based methods in untargeted metabolomics, with a focus on reporting on QC samples in sufficient detail for them to be understood, trusted and replicated. There is no intent to be proscriptive with regard to analytical best practices; rather, guidance for *reporting* QA/QC procedures is suggested. A template that can be completed as studies progress to ensure that relevant data is collected, and further documents, are provided as on-line resources.

**Key reporting practices:**

Multiple topics should be considered when reporting QA/QC protocols and outcomes for metabolic phenotyping data. Coverage should include the role(s), sources, types, preparation and uses of the QC materials and samples *generally* employed in the generation of metabolomic data. Details such as sample matrices and sample preparation, the use of test mixtures and system suitability tests, blanks and technique-specific factors are considered and methods for reporting are discussed, including the importance of reporting the acceptance criteria for the QCs. To this end, the reporting of the QC samples and results are considered at two levels of detail: “minimal” and “best reporting practice” levels.

**Supplementary Information:**

The online version contains supplementary material available at 10.1007/s11306-022-01926-3.

## Introduction

Progress in science is based on the principle that measurements are repeatable; that is to say, it should be possible for scientists in other laboratories, equipped with the same, or similar expertise, resources and infrastructure, to replicate the results of published work within acceptable levels of confidence. Properly documented studies allow other scientists to critically assess and judge the original experimental design and should also enable researchers in other laboratories to repeat the experiment under the same conditions and support or refute the findings. However, a reproducibility crisis in science has been recently highlighted by Munafò et al*.* (2017) and the lack of transparency in reporting can lead to ambiguity within experimental design, data collection, as well as data processing and interpretation, a situation which is also true for metabolomics. Moreover, any metabolomic data derived from instrumental analyses must be sufficiently reliable that decisions based on it can be reported with confidence; there should be no “leap of faith” from data to knowledge. If the metabolomic data cannot be trusted, then the answer to the posed question is of little value.

Quality management (QM) as a concept is very old and dates from at least the Middle Ages; the Assize of Bread in 1202 was the first English food law and outlawed the adulteration of bread with lower quality ingredients (Arayne et al., 2008). In the nineteenth and early twentieth centuries, QM became increasingly measured, systemized and subject to statistical rigor with the introduction of several food and drug laws (Arayne et al., 2008; Sarvari et al., 2020). QM can be divided into quality assurance (QA) and quality control (QC). According to ISO9000 (2015) QA addresses the activities the laboratory undertakes to provide confidence that quality requirements will be fulfilled, whereas QC describes the individual measures which are used to confirm and report that the requirements have been achieved. These definitions have been jointly endorsed by CITAC (the Cooperation on International Traceability in Analytical Chemistry) and EuraChem (A Focus for Analytical Chemistry in Europe) (Barwick, 2020). In general terms, QA processes are applied before and during data acquisition and QC processes are applied during and after data acquisition. These accepted terms for QA and QC are being used by the metabolomics QA and QC Consortium (mQACC) (Evans et al., 2020). More specifically, QA focuses on processes and practices that provide confidence that the end result will be of high quality. These processes are independent of, but can include, the data acquisition process. Examples of QA include standardized training, instrument system suitability tests (SSTs) and calibration, using documented high-quality and validated standard operating procedures (SOPs) throughout the study and performing regular lab audits. QC measurements are the measurements or observations undertaken to objectively demonstrate that the quality management process has been fulfilled. In practice, most QC measurements occur during or after data acquisition. Examples of QC measurements include, but are not limited to, analysis of QC samples such as reference standards, replicate extracted samples, pooled samples and blanks. Platform specific QC samples that demonstrate system suitability (sensitivity, freedom from artefacts, resolution, etc.) are also important in demonstrating the quality of the data generated. QC reporting is also more complete when reports include study-specific metrics reported in a clear, concise format. There are a number of similar terms in use for QA/QC in metabolomics that may cause confusion. A Glossary, where we have attempted to provide some clarity, is provided in this manuscript for reference.

The clear reporting of the analytical procedures applied, and transparency on the practices used in the laboratory, can provide other scientists with confidence in reproducing the resulting data or incorporating existing data into other studies, either directly (shared data) or indirectly (meta-analysis). Metabolic phenotyping studies typically begin with experimental design, and progress through sample collection, sample preparation, sample analysis and data collection. The process culminates in data processing and mining, which includes many different workflows depending on the question being addressed (Beger et al., 2019; Sumner et al., 2007). These considerations are of critical importance for the acquisition of high-quality, biologically meaningful data and full reporting can help to ensure confidence in the study (Goodacre et al., 2007). Appropriate reporting of the methodology used for sample analysis, including the pre-analysis procedures put in place to ensure quality and the operational procedures used to monitor the analytical process itself, enables data quality to be demonstrated and is intrinsically linked by QA/QC.

While many analytical QA/QC guidelines exist for *targeted* mass spectrometry assays (US Food and Drug Administration, 2018; European Medicines Agency, 2011), formal analytical guidelines for *untargeted* analysis are still to be developed [see e.g., (Begou et al., 2018; Naz et al., 2014)] and will then have still to gain widespread community acceptance. It would be of clear benefit to the field of metabolomics for QA/QC in untargeted metabolomics to be developed, supported, implemented and properly reported by the community. Such QA/QC reporting guidelines could be guided, perhaps, by the relevant existing standards from the FDA and EMA guidelines for targeted method validation (US Food and Drug Administration, 2018; European Medicines Agency, 2011). However, until such time as these analytical approaches have been agreed upon and accepted, there is no doubt that increased rigor in the reporting of how the QC sample results in reports and manuscripts were obtained is highly desirable. For example, the criteria used by authors to define acceptable performance in an untargeted analysis, together with the data used to demonstrate that this was indeed acceptable, should be provided in any publication or when data are deposited to public metabolomics data repositories. While we do not make specific recommendations for any particular approach or set of criteria for accepting analytical data, it is clear that such details should always be provided and be supported by readily accessible QC data.

Clearly a central argument for defining the quality of the information provided in any publication (or its supplementary information) and in data repositories is the ability to demonstrate that the QA/QC measures used before and during the analysis of study-derived (experimental) samples were both appropriate, suitable in terms of their design and study objectives and that the criteria used for acceptance were reasonable, achieved and reported. If the level of detail provided is insufficient for this to be assessed, it will seriously reduce confidence in results and therefore the value of the work. In fact, such factors represent a strong argument for journal editors and reviewers to suggest that the manuscript should not be accepted for publication in its present form. This may lead to requests for revision and resubmission or rejection. A secondary consequence of a focus on standardized reporting of QA/QC practices and results is the need to inform and educate those newly entering the field of metabolomics on the many factors that can affect data quality and influence results. A focus on QA/QC and dissemination of clear (minimum and best reporting practice) QA/QC practices should have the effect of naturally improving the quality of all metabolomics work.

Metabolomics researchers have undertaken efforts on standardizing reporting structures from early on in mammalian and plant studies (Jenkins et al., 2004; Lindon et al., 2005) which established SMRS (Standard Metabolic Reporting Structures) and ArMet (an architecture for metabolomics), respectively. These two groups subsequently combined forces with other international scientists and this led to recommendations for reporting standards in metabolomics, with the aim of identifying, developing and disseminating a consensus description for the best chemical analysis practices related to all aspects of metabolomics. This was nicely highlighted by the Chemical Analysis Working Group (CAWG) of the Metabolomics Standards Initiative (MSI) (Sumner et al., 2007) which was well received by the community at the time but has failed in its aims to be widely implemented (Spicer et al., 2017). More recently reporting standards for acquiring and analyzing untargeted and targeted metabolomics were suggested among best practice guidelines and relevant applications in regulatory toxicology by the MEtabolomics standaRds Initiative in Toxicology (MERIT) project (Viant et al., 2019). However, it is clear from even a cursory examination of the literature that these recommendations are often ignored. In addition, these references do not explicitly make suggestions for reporting of QC samples or QC results based on those samples. This manuscript proposes a framework for consistent reporting of QC sample information and quality metrics.

The need to report the quality of data measurements is more vital than ever as metabolomics-based research is increasingly used as a justification for large clinical or environmental interventions (Bearden, 2012; Gowda & Raftery, 2019). In that context, it is important to distinguish between reporting on the one hand and practice on the other. We wish to emphasize that here, we are not suggesting what constitutes best practice for QC in metabolic phenotyping, but rather ways to standardize what information authors provide in manuscripts to document the QC components of their work. Knowing that studies were performed in an appropriate way helps to ensure that the data quality is unambiguous so that others may replicate (or improve) the findings; we call this *Best Reporting Practice*. Many laboratories routinely carry out far more in the way of QA and QC processes than they report in formal communications. If the spirit of good scientific method writing is that another group can replicate the experiment, then the essence of QA and QC reporting must be that other scientists can properly judge the quality of the reported data as fully as possible and be able to replicate the same data quality guidelines.

Herein is a proposed set of minimal and best reporting practice standards for QA and QC practices for *analytical sample analysis* in untargeted metabolomics, focusing on the reporting of data from QC samples. Stages such as experimental design, metabolite identification, and other areas concerning data analysis to answer the biological questions/develop hypotheses, will be the object of future recommendations. Current recommended reporting requirements, for example, on “design of metabolomics experiments” have been presented (Goodacre et al., 2007). Given that much of this data is collected routinely anyway, the burden of reporting could be largely mitigated by the use of templates such that important data are filled in as it is collected. When studies are published, these could be included as a supplementary data file, with only the most important aspects included in the main body of the paper. A draft template that can be found in Online Resources (Table OR1 and a modifiable Excel spreadsheet based on it) that may provide a suitable model for describing the QC samples analyzed and representative quality metrics obtained in the course of a study. For the time-constrained reader, we have also offered a summary of the overall recommendations of this paper in Online Resources (Table OR1) and their justification (Tables OR2 and OR3).

## Quality assurance and quality control in metabolic phenotyping

For every stage (or segment) in the development and execution of a metabolomics study, there are important QA and QC steps that should be carefully performed and documented. Detailed QA/QC protocols need to be defined that, for *analytical data generation,* cover the following at a minimum: sample processing, use and preparation/sources of different types of QC samples, instrumental analysis methods, QC acceptance criteria and post-analytical data processing prior to data quality scrutiny and acceptance. Each segment of a metabolic phenotyping study has specific issues that need to be addressed by considering potential sources of error that would invalidate it, and the QA/QC measures adopted to prevent or reduce errors need to be reported in line with previously agreed-to standards. Here, as indicated earlier, the focus is on the analytical QA/QC processes based, most commonly, on either ^1^H nuclear magnetic resonance (NMR) spectroscopy or mass spectrometry (MS). MS-based analysis includes both direct infusion MS (DI/MS or DIMS) (which is essentially a form of flow injection analysis (FIA)) and separation-based MS techniques, including liquid-(LC) and gas-chromatography (GC) and capillary electrophoresis (CE). There are numerous other methodologies being developed for both MS and NMR, including imaging, direct analysis and in vivo modalities. However, currently the majority of published studies are based on GC–MS, LC–MS, direct infusion and high-resolution liquid state NMR spectroscopy and those modes serve as the basis for this report. As other approaches become more widely applied, the guidelines presented here should be adaptable enough to allow users of technologies not specifically covered here to report the quality metrics that were used with their analytical methods used.

In general, it is reasonable to expect that, whatever the method used, the analysis has been undertaken by a trained individual, that the procedure has been documented in detail (preferably with a written standard operating procedure (SOP)), that facilities are appropriate, the materials are of sufficient quality, and the equipment has been well maintained and appropriately used. Where SOPs are used it would clearly be of value to the community if, as best reporting practice, a version-controlled copy of the full SOP could also be supplied e.g., with DOI, Git or other accepted publication method. Reporting these details (and other QA aspects) as part of the manuscript QA section is crucial to strengthening the confidence of the report.

**Fig. 1:**
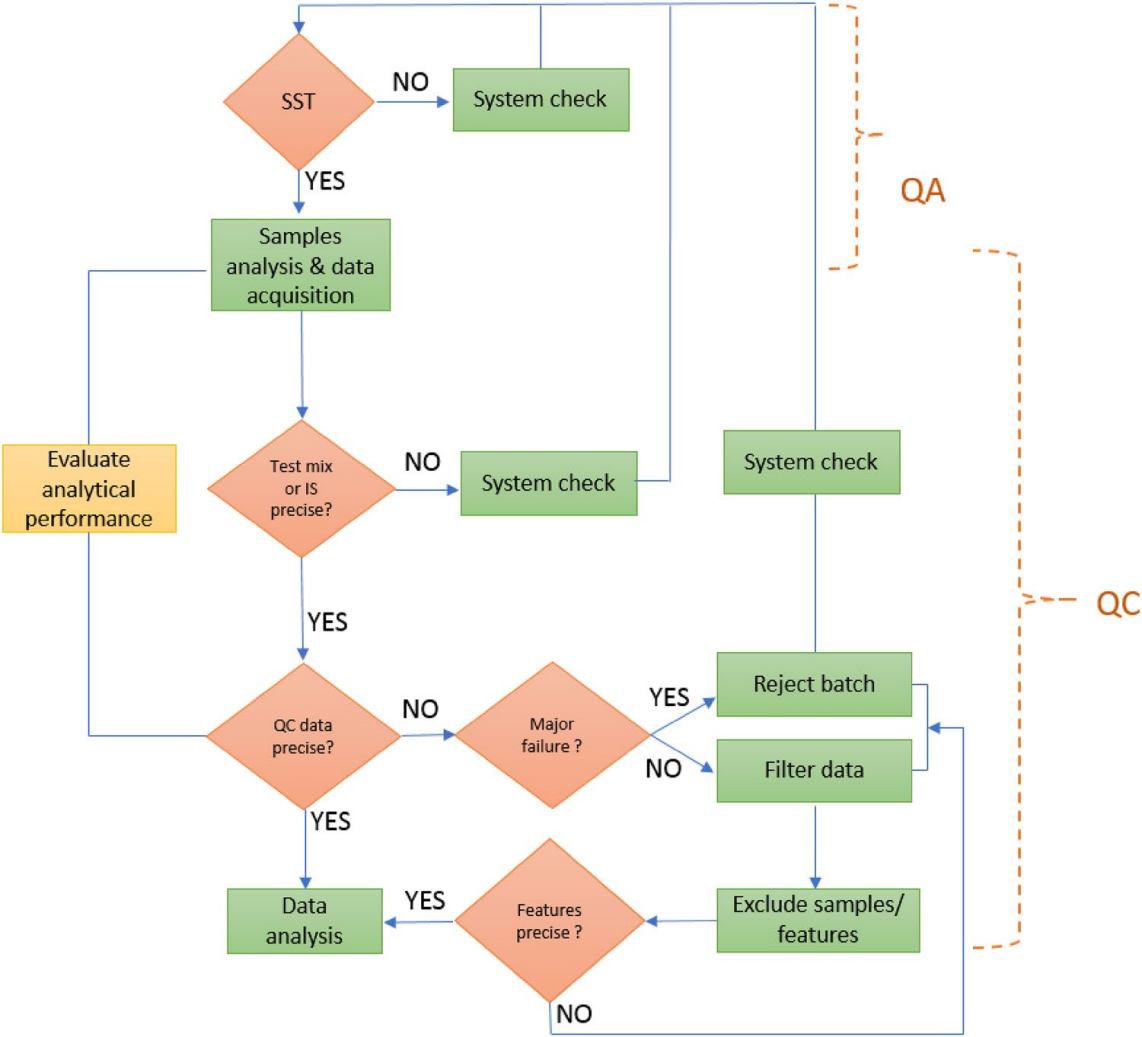
One possible QC scheme for the use of QC samples in the analytical section of manuscripts describing untargeted mass spectrometry based metabolic profiling. Clearly a similar one could easily be constructed that covers other methodologies such as e.g., NMR spectroscopy etc.

In targeted MS assays, where a specific analyte is measured, method validation gives an indication of the reliability of the data that will be acquired when the method is applied for the analysis of experimental samples. However, in untargeted profiling this is not possible as the analytical procedure aims to detect and measure an undefined number of metabolites, the identity of most of which are unknown. Despite this, it is possible to apply QC procedures that enable the continuous monitoring of measurement performance to demonstrate analytical precision or, on the other hand, highlights unexpected/unwanted deleterious changes. Having such data either allows verification of the analytical data, showing either that it met the quality requirements of the investigator, or did not, resulting in subsequent investigations to establish why the quality thresholds were not reached, and therefore what to do to improve the analyses. In Fig. 1 a QA/QC workflow that can be applied in untargeted metabolomics analyses is shown.

According to ISO/IEC 17025 “The resulting data should be recorded in such a way that trends are detectable. This monitoring should be planned. If the results of the analysis of data from monitoring activities are found to be outside pre-defined criteria, appropriate action shall be taken to prevent incorrect results from being reported”. It is important to emphasize that “appropriate action” should be taken which can include removal of data for specific metabolites or samples, or full re-analysis of samples or data. These guidelines can be adopted in untargeted profiling in chromatography-MS studies by setting up a quality control program that includes monitoring and documentation of, for example, the following: the type and number of QC samples used, randomization strategies, sample run order, number and frequency of QC samples per analytical batch, instrument parameter(s) monitored such as e.g., LC pressure curves, statistical analysis used for QC samples, illustrations used e.g. batch drift visualization, and control limits (Broadhurst et al., 2018; Dudzik, et al., 2018; Gika et al., 2016).

## Specific recommendations for reporting untargeted metabolic phenotyping studies

In recognition that in real life, science cannot always be perfect, the recommendations are divided into minimum reporting standards for QC data (the minimum that should be reported for a paper to be deemed of sufficient quality for publication), and “best reporting practice” standards for QC data (the reporting standards that experienced metabolomics laboratories should aspire to be routinely reporting).

**Fig. 2:**
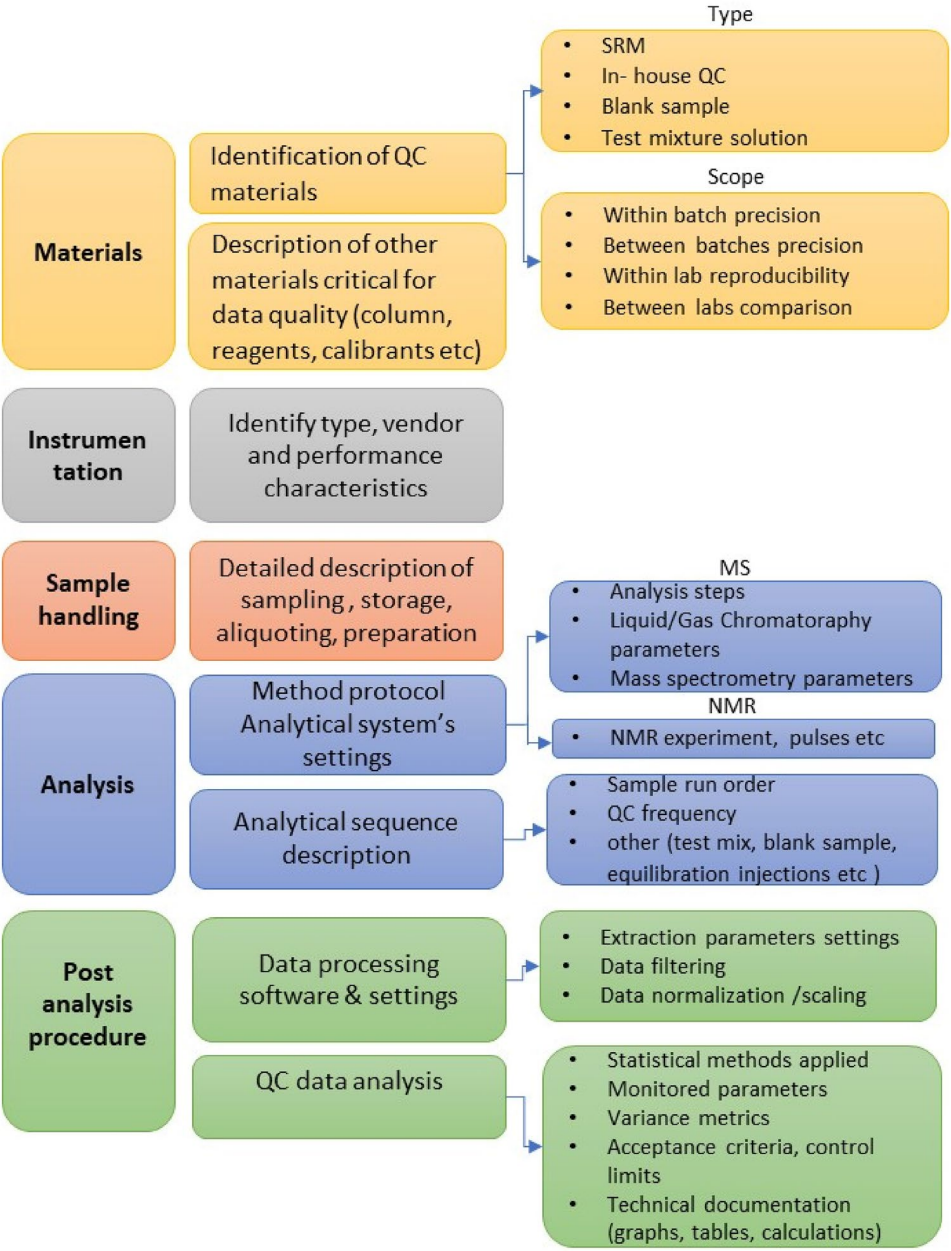
Information that should be documented in manuscripts to show the steps that have been taken to ensure the robustness of the analytical stages of a metabolic phenotyping experiment and its resulting data (see also Table OR3)

The type of information that should be included in the documentation of a typical untargeted metabolomics study must cover a series of factors that determine the reliability of the data. These factors and the recommended reporting items are described in detail in the following paragraphs. For ease, key reporting information, for what we consider to currently represent both minimal and best reporting practice, is listed in Table OR1 of the Online Resources while a schematic diagram of the suggested minimum reporting requirements is shown in Fig. 2.

It is recognized that it is sufficient to record many of the details below as part of the normal methods section of a manuscript. QC samples should be analyzed using the same analytical methods as the experimental samples, and where this is not done, the actual method used and the reason for the difference should both be reported.

### Quality control materials

#### Quality control (QC) samples

For both NMR spectroscopy and MS studies the type of Quality Control materials used should be identified and their purpose should be clearly described.

A number of similar QC sample types exist to enable the signals present in samples to be tracked over the course of the analysis for trends in properties such as e.g., intensity, retention time and mass accuracy (Broadhurst et al., 2018; Dudzik, et al., 2018; Gika et al., 2007, 2012). Based on the literature the most frequently used QC sample types are intra- and inter-batch pooled QC samples. There are also long-term QC samples to assess intra-lab repeatability or inter-lab reproducibility over a long period. These include inter-study QC samples to assess differences between separate studies on the same type of sample within one laboratory and inter-lab (between-laboratory) QC samples to assess differences between individual laboratories. No matter the way in which these matrix-matched QC samples are employed they have similar reporting requirements and are therefore considered together here.

The intra-study (within-study) matrix-matched QC samples, usually pooled samples, are meant to be representative of the samples being studied (Sangster et al., 2006). Pooled samples are used because of relevance, relative ease of preparation, economy and the acute shortage of suitable commercial matrix-matched reference materials. They are normally prepared by pooling aliquots of all the biological samples to be analyzed (although if this is impractical it may be a “representative” selection, such as all the samples on a particular 96 well (or higher) plate, for example). Increasingly, laboratories are also running additional *phenotypic* intra-study QC samples (i.e., samples where each class is pooled separately). Phenotypic QC samples might be used to highlight differences in precision between experimental groups (especially in statistically unbalanced studies) vs control samples for signals differentiating between the classes (e.g., healthy vs disease). This might be the result of e.g., analytes having been “diluted out” in the study pool or are perhaps even entirely absent in one sample class, or so strongly elevated in concentration that they saturate the detector. Intra-study QC samples can be used in a single study batch (intra-batch) or across several batches from the same study (inter-batch) to enable the results for different batches of samples to be compared. Inter-batch QC samples may be used in short term studies that require several analytical runs to be performed over a few days, perhaps with preventative instrument maintenance carried out between runs. They may also be used by different laboratories that are collaborating on inter-laboratory trials. “Long Term Reference (LTR)” samples are analyzed across multiple batches often across multiple studies and are normally composed of bulk matrix samples (e.g., pooled plasma or urine from healthy donors). LTRs are generally used where many batches of samples, perhaps in several collaborating laboratories, are being assayed using the same methodology and there is the desire or intention to compare them or even to correct them (Bijlsma et al., 2006; Kamleh et al., 2012; Lewis et al., 2016; Zelena et al., 2009) to ensure comparability. As such, LTRs do not necessarily replace “conventional” study derived QC samples because they may not be strongly matrix-matched. Such LTRs are currently generally prepared by those laboratories where they are employed or are purchased from a commercial supplier; for example, the NIST human plasma SRM 1950 could be used as an additional QC to facilitate comparison across multiple labs and studies. Whatever their source, assuming that they are consistent, LTRs act as a fixed comparison point between studies, and are ultimately the final arbitrator of data quality and comparability. The various types of QC in metabolic profiling studies described above are summarized in the Glossary and, in more detail, in Table OR 2.

**Fig. 3:**
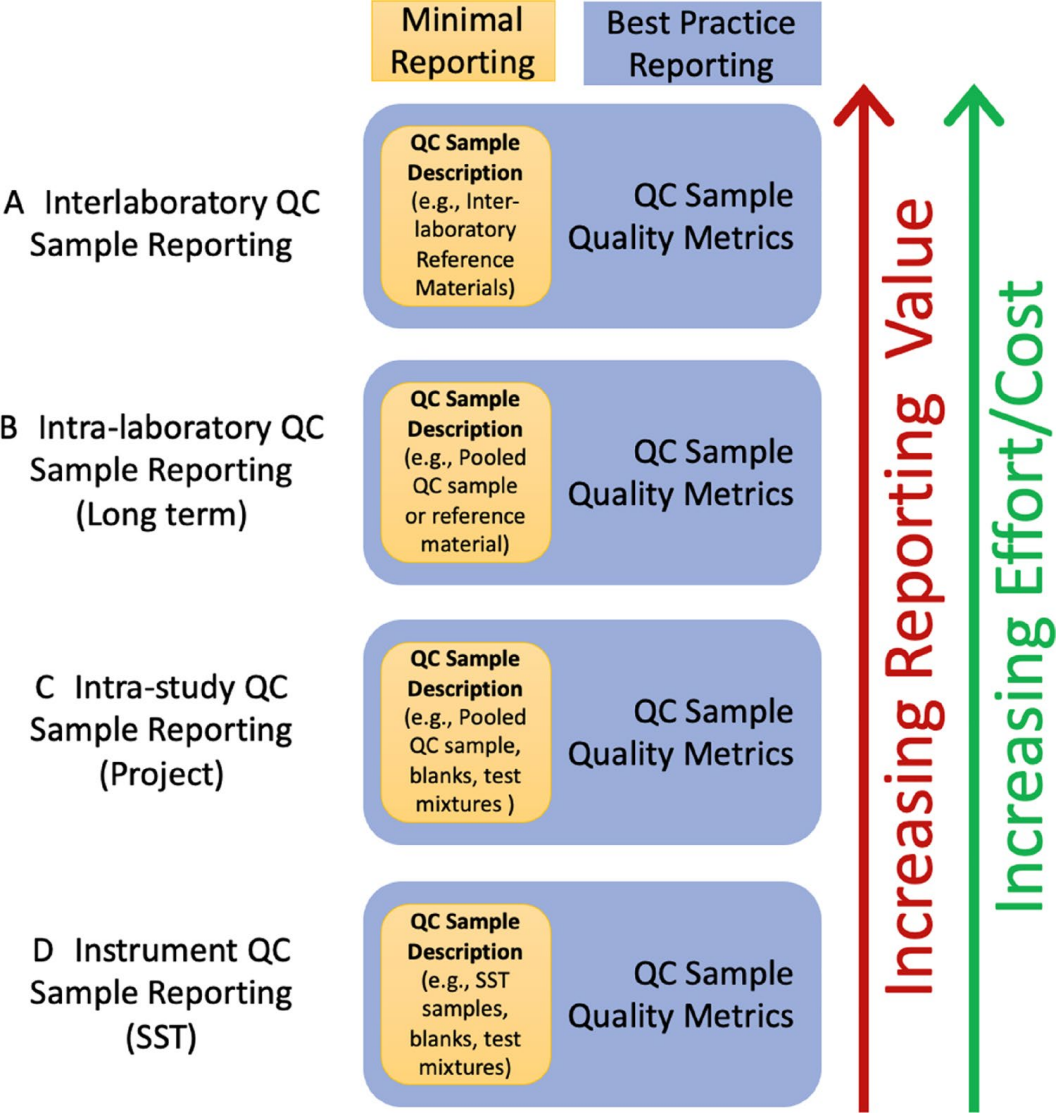
The various cumulative levels of analytical reporting for QC samples are depicted in a hierarchy of value and effort. Each layer builds upon lower layers. **A** The samples that support interlaboratory comparability have the highest value and can be reported in the minimal sense (a qualitative description of the samples in the study) and in a Best Reporting Practice sense where QC metrics are also reported. **B** Long-term intra-laboratory QC samples represent ongoing efforts in the reporting laboratory to present consistent results across their various projects, and these can also be reported in a Minimal or Best Reporting Practice sense. **C** Individual project comparability during the analytical phase of the project can be demonstrated by Intra-study QC samples and reported in a minimal or as best reporting practice. **D** Instrument QC sample reporting demonstrates fitness-for-purpose of the instrument at the time of the project and represents the foundation upon which the other layers rest

Clearly, the fact that the use of QC samples is now considered standard (Beger et al., 2019; Broadhurst et al., 2018) represents a welcome advance in analytical practice, but there remain wide variations in reporting. Reporting more typically involves more work, but increases the potential gain (Fig. 3). Irrespective of the type of QC sample(s) incorporated into the analysis, it is incumbent upon the authors to report what they were, how they were used and the criteria for acceptance. Thus, where QC samples have been used, we recommend that, as a minimum, the following should be reported:

- Sample matrix type: specific cell type, tissues, body fluids, etc., including species of origin and details of collection if different from experimental samples. Any preservatives or additives (e.g., anticoagulants) which were used in the collection or storage of the samples should be reported.
- Origin: *If from experimental samples*, which experimental samples were pooled to create the sample. For example, all or a subset of samples, of the same study or of a previous study. *If commercial*, the supplier information, any additional metadata known, product number, and batch/lot number.
- Storage and Preparation: Storage conditions and methods used to prepare the QC samples are important and should be reported. This should ideally include aliquot volumes, freeze/thaw cycles, and details on QC preparation. For example, assuming a sample preparation step, how were the QC samples collected and processed before extraction? Extraction of multiple individual QC aliquots enables the assessment of both extraction and instrumental variability. Conversely, if the QC was pooled post extraction i.e., one extract was injected multiple times, then only instrument, but not extraction, variability can be assessed. For this reason, it is important to note the preparation stage at which the sample was pooled. For example, if plasma samples were aliquoted to form the QC before any sample preparation or following protein precipitation.
- Scope of use: The intended use of samples should also be reported. This includes whether the QC samples were used to assess the precision within a batch, or the precision between batches, or for signal correction, or to compare results from a different study within one laboratory/ between laboratories or a combination of these, etc. More details of potential uses are provided in the analysis section.

#### Internal standards (IS)

Internal standards can also be used for QA/QC purposes e.g., for monitoring chromatographic drift or intensity over time or for assessment of NMR spectral quality. If used, information regarding the use of IS in either QC or in the experimental samples should be reported. This includes the concentration used for each analyte and the stage of addition (in the original samples / QC or in the extract at any stage prior to analysis). The source and purity of the internal standards should be reported.

#### Blanks

It is important to differentiate between process blanks and “solvent blanks” or “true blanks”. True blanks are mobile phase (LC) or background electrolyte (CE) which are analyzed independently, i.e., some form of dummy injection is made into the instrument. Process blanks are water or solvent samples prepared and treated exactly like experimental samples, including undergoing any extraction processes. They can be used to highlight contaminant peaks arising from vessels or chemicals used in sample processing. Both Process and True blanks will detect “system” peaks, such as mobile phase contaminants. Analyzing both enables the source of the contamination to be isolated. All blanks can also be used for detecting and quantifying sample carryover. Minimum reporting standards for blanks should include:

- The type of any blanks (solvent blanks, process blanks etc.) used and how they were prepared.
- Procedures and methods used to prepare the process blank QC sample, including vial-types used and volumes of aliquots, especially if these were different from those used on the experimental samples. Details on solvents including the supplier and their purity should be included, as should the use of any additives such as buffers etc., and sodium chloride solution (where biphasic extractions are used).
- Acceptance criteria should be reported including e.g., the acceptance criteria for peaks which appear in the blanks or maximum acceptable carryover

#### Test mixture solutions

If a synthetic mixture comprised of chemical standards is used, its composition, concentrations of the compounds used and, importantly, method of use, should be reported.

If this mix is used as an SST, minimum reporting practice requires reporting:

- For laboratory-prepared solutions, individual components of the standards mix, their concentrations and, ideally, the source and purity of each chemical.
- For commercial test mixtures/reference materials, report the product name and source, and product number etc.

#### MS-based platform(s) specific

Whilst perhaps not strictly related to sample QC, information that is material to the analysis should be provided for LC, GC or CE systems coupled with mass spectrometers. Minimum details would include the type(s) of chromatographic column used, its source and its characteristics, unless it is being used in a propriety method. For GC, the derivatization reagents used should be reported, and whether this was performed in batches or using just-in-time derivatization automation. Mass calibration standards and other materials used for sample cleaning or treatment should be provided if considered critical for the acquired metabolite fingerprint (solvents, salts, sorbents, vials etc.).

#### NMR spectroscopy specific

The preparation of QC samples for NMR spectroscopy studies is not too different from MS-based studies. But because there is no requirement for multiple internal standards for identification or quantification of analytes, any internal standards used should be carefully described and quantified. Typically, a bulk supply of solvent is prepared for a study for rehydration or dilution of experimental and QC samples immediately before analysis. The careful preparation of this bulk solvent, which may include a buffer, is crucial for quantification and replication across studies, so details of preparation should be reported. Minimum reporting should document the solvent, for example 10% D_2_O/90% H_2_O or 100% D_2_O; it is rare to use other solvents in biological metabolomics studies, but in lipidomics less-polar solvents such as CDCl_3_ are often used. The chemical shift reference compound is usually added in this bulk solvent preparation to ensure uniform concentration across all the samples in the study, but if this is instead added as an extra step in the sample preparation from a concentrated stock solution, it is a crucial difference which must be reported clearly because the inherent errors in adding small volumes will affect any subsequent quantification. There are several chemical shift reference compounds commonly in use in NMR spectroscopy-based metabolomics, so it is crucial to positively identify which compound is used and its labeled deuteration level. Rarely, a project may include pH-indicating compounds or additional quantification internal standards; these should be fully described (name and concentration) in the QC report. Any analysis of the prepared bulk solvent by NMR spectroscopy (or other means) for impurities and suitability should be reported, noting whether this analysis was performed before any experimental samples were rehydrated, or diluted, with the bulk solvent to detect any inadvertent contamination.

The type and source of the NMR tubes used for the experimental samples and QC samples should be identified by manufacturer part number in the QA/QC report. If disposable, single-use NMR tubes are used, the QC report should indicate any contamination detected in solvent blanks run in the batches as there may be residues from the manufacturing process. If NMR tubes are recycled, the details of the tube cleaning and drying process should be reported as well.

### Instrumentation

The instrumentation used to perform sample analysis is clearly critical to the type(s) of data that can be obtained. The minimum information that should be supplied is detailed below.

#### Type, manufacturer and software

Type(s) of mass spectrometer (e.g., quadrupole-TOF MS, quadrupole-Orbitrap MS etc.,) or NMR spectrometer field strength(s) as well as the model, software, and manufacturers details must be reported. Similarly, if hyphenated to a separation method, clear details of all instrumentation should be provided including any sample handling robots or similar preparative equipment.

#### Calibration

The method of instrument calibration, tuning, and details of any test compounds/mixtures used, and their origin should always be provided to demonstrate that the devices are operating to the specifications of the manufacturer. As part of *best reporting practice* data for MS tuning and calibration, mass resolution and accuracy should be provided, and the frequency of calibration should be clearly stated in the manuscript. As discussed earlier, if possible, chemical identity, parameter(s) and acceptance criteria for each calibrant should be stated.

For NMR spectroscopy, temperature calibration and stability are crucial to obtaining consistent results such as high-quality and consistent water suppression and consistent spectral linewidths and positions. Using standard protocols (Findeisen et al., 2007) it is feasible to obtain temperature stability of at least 0.1 ºC at the probe and, if this has been done, the method of sample probe temperature calibration should be stated. In addition, the probe used should be characterized for the amount of time taken to reach temperature equilibrium after sample insertion; this may range from 1 to 10 min, depending on the probe construction and the temperature controller used. This equilibration time should be noted in the QC reporting documentation.

In addition, calibration of the NMR chemical shift reference material is often set to a specific value automatically, and if so, this value should be noted.

#### System suitability testing (SST)

SST includes all those tests which are carried out on an instrument prior to the analysis being performed. They are designed to ensure that the instrument is working correctly and that it reaches the minimum performance requirements for the analysis.

For MS-based methods, SST include regular instrument checks and specific tests, such as the analysis of test mixtures or samples to check for instrument calibration, including the examination of background noise, mass accuracy, and mass resolution, signal intensity etc. When a separation technique is used in combination with MS then retention or migration times, peak symmetry, peak responses (height or area) and chromatographic resolution should be determined to ensure that they are within the predetermined tolerances required for optimal performance.

Whilst more of a QA procedure rather than an SST, there may also be value in noting any other interventions that are critical for the quality of the data acquired such as maintenance cycles, routine column cleaning and equilibration, injection system, temperature and duration of column baking out etc.

For ^1^H NMR spectroscopy, system suitability testing is very closely related to instrument performance acceptance procedures which should be common practice for NMR spectroscopists. Details of the specific instrument tests performed must be reported as part of minimum reporting practice. A few examples of the tests that are necessary to demonstrate suitability of an NMR instrument before a project begins are: water suppression experiments, signal-to-noise assessments and temperature calibration experiments. While other approaches may be chosen, often these tests can all be addressed by meeting instrument specifications for a 2.0 mM sucrose sample in 10% D_2_O/90% H_2_O standard sample (ASTM International, 2015). The proper execution of a water suppression pulse sequence on this sample can be used to demonstrate that the quality of shimming, peak separation resolution, linewidth of the suppressed water resonance, ^1^H pulse power calibration and spectral signal-to-noise ratio etc., shows that the instrument performance was suitable for acquiring spectra. The qualitative and quantitative results from a system suitability test using the sucrose sample, or other suitable samples, should be reported in the QC section of the report. System suitability is often performed during the batch runs on the included QC samples and on the experimental samples via automated scripts that evaluate spectral performance. Under automation, it is possible to assess the linewidth for each sample and also calibrate the ^1^H pulse duration for each sample. If abnormalities are detected during the individual sample analyses, it is possible to rerun the sample, paying special attention to the automated shimming of the sample. The criteria for decisions on whether or not to rerun a sample and the timing of the rerun should be reported in the QC report. The average linewidth of the internal standard, signal-to-noise of the internal standard and pulse calibration width for the batch or project should be reported in the QC section of the report to demonstrate consistent performance of the instrument during the instrumental analysis phase of the project.

SST reporting standards for all instrumental techniques should minimally include:

- Analytical methodology of the SST
- Solvents, samples and analytical methods used (especially if deviating from the manufacturer’s recommended methods and/or if the methods are different from the method used for the analytical run)
- Amounts/volumes of test materials used for analysis, including any additional information such as injection volumes, split ratios, etc.
- Results of any SST performed, utilizing pre-defined acceptance criteria and whether the acceptance criteria were met.

### Sample handling

Sample collection and handling are important areas of QA and are too involved and complex to fully cover here. They will form the subject of a future paper. However, since sample handling may clearly affect the QC results (or their interpretation), it is covered very briefly here to note that sample handling protocols and any deviation from them should be well controlled and documented. This includes any procedures occurring after the samples have arrived at the lab, including freeze/thawing, subsampling of single aliquots, aliquoting, storage conditions, thawing conditions for sample preparation, etc.

Minimum reporting requirements for sample handling include:

- Metabolism quenching protocol
- Storage conditions including temperature, duration and aliquot size of samples and QC samples.
- Thawing conditions
- Aliquoting scheme
- Sample preparation protocols applied to experimental and QC samples (volumes, solvents, procedures, extraction, derivatization, centrifugation, filtration, and other critical details such as GC–MS or LC–MS batch preparation and sample autosampler queue time). Typically, the same procedure is applied for QC and experimental samples.

The number of freeze–thaw cycles for experimental samples should be provided as best reporting practice. If a large number of samples are prepared all together or as batches and stored till the analysis or are analyzed directly and remain under the autosampler conditions for long periods of time, this should be stated. In GC–MS analysis protocols where derivatization takes place, time intervals between sample prep and analysis are often critical. Data that provide proof of sample integrity during sample treatment or analysis are desirable in these cases.

### Analysis procedure

As different procedures are followed depending on the applied technique, reporting requirements are listed for LC–MS, GC–MS and NMR spectroscopy platforms separately.

#### MS-Based platforms analysis

(a) Method details

For minimal QC reporting, all critical details (instrumental and method parameters) that affect the analytical results should be reported unless it comes to a proprietary methodology where only selected information can be disclosed. In such cases, there should be a justification of why all method details are not given, and the QC results should be fully reported to enable readers to assess the method’s fitness for purpose. As new instruments are continually developed, it is difficult to present a comprehensive reporting list without it quickly becoming out of date. Guidelines for reporting have been presented here, but individuals should report all methods in enough detail that another individual can repeat the analysis exactly on the same model instrument. Reporting items should include the chromatographic column, type, dimensions, particle type and size, manufacturer, elution program (solvent compositions or thermal gradient), flow rates, injection volumes, split ratios, temperatures, source parameters (electrospray voltages, gas composition and flow rates, temperatures and additional instrument-specific information important for repeating the analysis), MS and MS/MS detection parameters (*m*/*z* scan range, resolution, scan rate, collision energy(s)), additional specialized parameters e.g., for MS^E^ acquisitions, 2D mass spectrometers, or for IMS, lock mass calibrant used in line/offline for TOF MS systems, etc.

(b) Analytical sequence details

The full and accurate description of the overall plan of analysis to assess the data quality during, or after, the analytical run represents a minimum reporting standard. This description should include the sample run order, (i.e., the order in which individual specimens are analysed, including where various blanks and QCs samples have been placed in relation both to each other and the samples being profiled), the number of samples per batch, the duration of the analysis and the total number and frequency of QC injections. It is generally accepted that, as a minimum, 5% to 10% of the samples analyzed should be QC samples, but the frequency of QC analysis should be justified by the number of experimental samples, instrument technical variation and the intended use of the QC samples. In addition, the following represent minimum reporting standards: The number of replicates per QC aliquot should be provided; for each individual analytical run, the number of individual QC samples and the number of injections from each vial should be given.

- How many QC samples were prepared and analyzed? What was the frequency of analysis? Was more than one type of pooled QC used? e.g., phenotypic or long-term QC samples etc.
- Where “column conditioning” was undertaken conducted at the beginning of the run how was it performed. Often column conditioning is performed using a number of injections of a QC sample and the number of sample injections (and the volume used) should be reported. The acceptance criteria used to assess that the system was equilibrated based on the column conditioning should be reported.
- It is generally accepted that, whilst the QC samples are analyzed at regular intervals, the study samples should be analyzed in a randomized order. If samples and/or QC samples were randomized, the method of randomization should be reported.
- If multiple injections of test samples were made for QC purposes, then this should also be described.
- What number and nature of blanks analyzed were used?, and in which order? Was each aliquot analyzed once, or was repeated use of the same blank aliquot used? How many blanks were prepared and analyzed?
- Number of synthetic mixtures of metabolites /test mix solutions analyzed, in which order and with what frequency?
- Acceptance criteria for QCs should be provided, and confirmation that these criteria were met.

For best practice reporting, the time period of a non-stop analytical batch, as well as the time interval between different batches for samples from the same study could usefully be reported. In addition details of scheduled or other maintenance of the system which occurred between batches from the same study, e.g., cleaning of the source, mass calibration, rinsing or exchange of the chromatographic column in LC, “bake out” in a GC system or the exchange of separation capillary in CE etc., represent useful supporting information on the conduct of the analytical aspects of the study.

#### NMR Spectroscopic analysis

- Method details

Details on how the QC samples and experimental samples were analyzed for spectral quality during the data acquisition phase should be reported. State whether or not each spectrum was assessed for QC metrics such as: linewidth, pulse width, baseline roll and water suppression quality. Clear details of how the spectra from different samples were collected should be provided such as whether or not this was done with fixed instrumental parameters (like receiver gain or pulse widths) or if these features were changed from sample to sample. It is desirable to report the range of times between solubilization of the sample and data acquisition because of the potential of sample degradation. Best reporting practice would be to describe whether or not sample spectra were examined for spurious contamination issues. Because sample stability can be a concern when a sample has to be reanalyzed as a result of spectral quality issues or sample degradation then this should be indicated. If re-analysis occurred some statement as to the timeframe for re-analysis is necessary. For example, was re-analysis performed as soon as possible after the issue was noted? or at the end of the whole experiment? Alternatively, the decision might be taken to delete this sample from the data set in which case it would be useful to indicate the criteria on which the decision was based.

Often additional spectra (e.g., pseudo one-dimensional or two-dimensional experiments) may be collected to aid in compound identification, so these auxiliary experiments should be described and details about whether these spectra were collected on every experimental sample or on a representative set should be stated.

(b) Analytical sequence details

Additional analytical details that affect the batch data quality should be given. For example, is there evidence that the water suppression was consistent throughout the batches or the whole project? Was shimming of the magnet acceptable for each sample? Was the NMR spectroscopic analysis continuous for the project, or were batches run in a discontinuous manner over an extended time period?

### Post analysis procedures

The procedures that follow instrumental sample analysis should be described, including all details of the processing of the LC–MS, GC–MS, DIMS, CE-MS or NMR spectral data into data matrices used for quality control analysis. The suggested reporting here applies only to the data analysis performed up to the point that the quality of data was shown and documented. It should be stressed however, that reporting requirements associated with the data analysis stage of a metabolomics study have been extensively described previously by Goodacre et al. (2007). In general, enough detail should be provided that another scientist could repeat the analysis.

#### Data extraction

For MS based platforms, minimum reporting would include the software used (for all platforms), its source (vendor, version, for in-house or open access references), and some detail regarding the following parameters: deconvolution setting, peak picking and chromatogram spectral alignment, mass tolerances and retention time thresholds, abundance thresholds, signal to noise ratio, retention time correction as well as noise filtering settings, and parameters for isotopic masses and adduct-clustering. These settings differ in the various software packages however, this information can aid the reproduction of the data from a similar system and provides a general overview of data processing conditions as these are roughly similar irrespective of the algorithm applied. Since default values may change over time or with different software versions, individuals are encouraged to report the actual values used.

For NMR spectroscopy minimum reporting should include the software used in preparation for multivariate (or other) analysis. The details of post-acquisition processing for spectral quality such as any applied signal processing, baseline correction, phase correction or signal enhancement should be provided. The parameters used in data post processing prior to chemometric analysis should also be reported including spectral alignment, binning mode (if any) or feature selection parameters, and finally, any normalization (total spectral area, for example, or specific spectral features selected for normalization), scaling and transformation applied. All spectral regions removed (e.g., residual water region, urea region in urine, etc.) should also be reported.

For both MS- and NMR-based data sets, if features are excluded based on threshold settings dictated by signal-to-noise ratio, or the frequency of their presence in samples, blanks or QC samples, or those which are removed based on the variance of their intensities in the QC samples, these should be minimally reported as they affect the dimensionality of the final exported data matrix. For the reporting of QC data, whether the data processing applied was pre- or post-normalization it should be defined as this can influence QC acceptance criteria. It is also the case that sometimes row/column scaling or normalization of the data is applied based either on total signal or mean intensities of IS's or specific endogenous metabolite peaks. Information on these data transformations should be included in all manuscripts as they represent minimum reporting practice.

Best reporting practice would encompass not only the processed data being made available on publication but that the raw data is also made accessible for all samples analyzed (both study and QC samples). This is suggested with the caveat that there are circumstances where immediate release of such data may not be possible but where embargoing may be appropriate.

#### Quality control analysis

The aim of expending time and effort on QA/QC procedures is to have confidence in the robustness of the analytical results. This section is normally reported in the results section and enables readers to judge the analytical success of the analysis. Disappointing figures here do not immediately disqualify results from being meaningful, but extra scrutiny should be applied by both the author and the readers to assess the validity of any biological conclusions. Data should only be excluded based on objective QC performance or on sound statistical practices. Any excluded data should be clearly reported. The data quality analysis can include various steps from inspection of chromatographic traces (LC/GC–MS) or spectra (NMR) to statistical analysis of acquired QC data. For MS-based platforms minimum acceptable reporting should include:

- The predetermined acceptance criteria for a series of samples within a run based on test mix, blanks and QC analyses.
- The peaks monitored from test mixtures or QC samples, target ion selection parameters, mass range, parameters examined (peak areas, mass accuracy, retention time (t_R_)).

For NMR spectroscopy-based platforms minimum acceptable reporting should include:

- The predetermined acceptance criteria, such as spectral median standard deviation (Parsons et al., 2009), for a series of samples within a project based on QC samples.
- The typical (or averaged) signal-to-noise for the experimental samples based on an added internal standard compound, the typical (or averaged) proton pulse width and the typical glucose resolution (if the samples contain glucose).

Irrespective of the technique used, minimum reporting of data quality should include:

- **Monitoring of random or systematic effects, including intra and inter batch effects**. If the QC samples have been used to monitor random or systemic effects it is important to report how this was done e.g., where Principal Components Analysis (PCA) was applied, which samples were excluded? and which combination of PCs were used? If plots of selected ions against time/injection order were used to obtain a preliminary assessment of the data quality (e.g., platform stability and obvious run order effects) the outcome should be reported. If such run order effects were seen by PCA, was further action via e.g., Time Series analysis etc., undertaken to explore them? Any action taken should be described, and the results reported. Ideally, where a numerical method was used alongside a visual method to monitor run order/batch effects, e.g., calculating the sum of squares (SSQ) deviation from the horizontal etc., the results should be reported.
- **Precision of the analysis** is based on the results for the QC sample analysis. Authors should define how these were calculated (i.e., were all relevant derivative or adduct peaks used individually or summed together, or was only the most intense one used, how were missing values accommodated and how were data normalized, and missing values handled, when calculating RSDs for the QC data, etc.).
- **Precision of detection** is based on QC sample analysis. This is indicated by reporting the number of QC samples in which an individual metabolite or signal was detected.
- **Subjective proof of data quality** based on illustrative examples of the parameters monitored such as plots showing batch drift evaluation (intra- and inter- batch including significant drift in retention time or change in response) e.g., see (ASTM International, 2015). Different metabolites can have different degrees and directions of batch drift over the course of an analytical sequence, and this should be reflected in the reporting data. No specific metrics have been determined for minimal reporting, but authors may wish to consider modelling the intensity trends, both intra-batch and/or inter-batch and reporting trend lines, standard deviations, etc., as well as providing graphical presentations and a PCA that includes the QC samples etc.
- **Acceptance criteria** for sample and feature inclusion and the numbers of features considered should be provided as a minimum.

Irrespective of the technique used, Best Reporting Practice of data quality should include:

- Intra- and inter-batch QC RSDs for all reported compounds/ features.
- Objective numeric data should be provided to assess the degree of batch response or RT drift (a PCA plot alone is not deemed sufficient in itself to prove that batch drift was minimal, although it can be used alongside other relevant data)
- Details including name, version number and computer environment of any software package or algorithm used to correct batch drift including the parameters used for the algorithm (e.g., order of spline if spline correction used).
- If a newly developed algorithm is applied for data quality assessment or correction/improvement based on QC series data, it should be described, and references should be provided (if not published it would be best reporting practice to provide the code in e.g., supplementary information or on Git repository hosting services).

A description of all key reporting features and the rationale behind them is summarized in Table OR1 of the online resources, and an excel spreadsheet that can be updated as studies progress is also provided in the OR.

## Discussion and conclusions

The use of both QA and QC procedures is recognized as fundamental for achieving reliable and consistent data in metabolomics. The recommendations presented here are not meant to constrain research but to ensure that, where these systems have been employed, this is stated and what has been done is clearly explained. In this opinion paper there is no official authority to mandate the procedures that must be used when employing QA/QC in untargeted metabolomics. However, the recommendation here is that authors state what they did, and to state this as transparently and as completely as possible. In other words, they should deliberately and purposefully avoid obfuscation. The documentation of the QA and QC of the analytical process of an untargeted metabolomics experiment enables experimenters to demonstrate that the work was conducted appropriately, and that the editors and reviewers, and therefore ultimately readers can have confidence in the resulting conclusions. However, recognizing that untargeted metabolic phenotyping is a discovery methodology any hypotheses emerging from it are inevitably provisional. Nevertheless, armed with knowledge that the data are of good quality provides the motivation to devote the resources required to undertake further targeted analysis, using quantitative and validated methods, to confirm, or refute, these hypotheses.

These recommendations will, no doubt, be modified as practices evolve and there will be future discussions on other important topics such as sample collection and storage, as well as data processing practices and their robust reporting. For such measures to be widely adopted requires consent and buy-in from the metabolomics and wider community. Towards this end, a number of virtual meetings are planned within twelve months of this publication to enable all interested parties to debate the points raised here and a final community consensus to be reached. The meeting dates and times will be published on the mQACC website: https://www.mqacc.org/

## Glossary

**Table:** 

Term	Definition
Precision	Performance characteristic of the analytical procedure that is applied to produce the untargeted spectral profiling data. It indicates the typical measurement variability of the repeated measurements of the same sample within a time period of a batch (repeatability) or over longer periods e.g., between batches (intermediate precision) and over a long period of time (intra-laboratory reproducibility). It can be assessed by designed replicate analysis of QC samples under the operational conditions and it is usually expressed by statistical parameters which describe the variability of the data e.g., the standard deviation or relative standard deviation of signals. Accuracy is not the same as precision as, unlike the latter, it cannot easily be determined in untargeted MS-based profiling as the identities and quantities of the solutes in the sample are not known prior to analysis.
Mass accuracy	Mass Accuracy is a measure of how close the measured mass of a standard calibrant is to that obtained when measured experimentally. It is an important measure of mass-spectrometric instrument performance.
Intra-laboratory reproducibility or Within-laboratory reproducibility	Indicates the variation in the analytical data if the same sample is analyzed in the same laboratory at different times. It encompasses the whole analytical process from the sample entering the laboratory to the report.
Inter-laboratory reproducibility or Between-laboratory reproducibility	Indicates the variation in the analytical data if the same sample(s) are analyzed in different laboratories.
Batch	The collection of experimental samples, and QC samples (including, but not limited to blanks, experimental sample replicates and other pertinent quality-related samples) from a study that are processed at one time and analyzed in a single, non-stop instrumental run.
QC sample(s)	Various types of mixtures prepared to assess the quality of untargeted data.
Pooled QC sample	A matrix-matched QC sample prepared by mixing aliquots from all the samples of a study. In the case of large studies, it can be prepared from a subset of the samples. Various types of pooled QC can be prepared depending upon their intended use (see below).
Phenotypic pooled QC sample	When a matrix-matched phenotypic QC for each class of samples under study are prepared separately. It can be used to highlight differences in precision in test vs control samples for signals differentiating between the classes.
Intra-study QC sample (Including Intra-batch/Within-batch QC and Inter-batch/Between -batch QC)	A QC sample that is used to assess the precision of untargeted data along a single batch (intra-batch QC sample), or a single study (inter-batch QC sample). It can be a pooled QC prepared from aliquots of the samples analyzed in the batch itself (see above) or, where this is impractical, other approaches (e.g., a “bulk” QC sample of the same matrix) can be used.
Long term QC (can be subdivided into intra-lab /within-lab and inter-lab/between-lab)	A reference material or a bulk QC sample prepared from a set of samples, or a bulk sample/reference material purchased from a public source. It can be used to assess analyses undertaken over a relatively long period for intra- and inter laboratory reproducibility. It can be used to assess (and potentially correct for) any differences between separate studies on the same type of sample. Intra-laboratory (within-lab) QC samples are used only within a single laboratory. Inter-laboratory (between-lab) QC samples are used to compare data between two or more labs.
Blank(s)	Blank QC samples are important in demonstrating process purity, where extraneous signals are identified and accounted for. A true blank is a neat solvent/buffer etc., with minimum pre-processing, that is directly analyzed by the instrument. If the sample is a buffer aliquot, for example, it could be either a process blank or a true blank, depending on how it is handled. A process blank consists of a neat solvent, water, buffer etc., sample processed in exactly the same way as the samples. It can be used to examine the interferences or contamination introduced by the analytical system, columns, vials or the sample preparation step.
Test mix(ture)	Often a synthetic mixture of metabolites, including representatives of the metabolites expected to be in the study samples. It can also be a mixture of exogenous compounds or xenobiotics that are easily or often detected in the analytical system used.
System suitability test (SST)	Analysis(es) performed before the analytical batch of the study samples to check that the analytical system is working appropriately and fit-for-purpose. It can be performed using QC samples or with a specific standard mixture (see above).
Reference materials	A reference material is a material which is sufficiently well characterized, homogenous and stable to be fit for its intended use in a measurement process. Reference material is a generic term. A certified reference material (CRM) has been additionally characterized by a metrologically validated procedure for specific characteristics which will be documented in an accompanying certificate including the allowable measurement uncertainty. A standard reference material (SRM) is a reference material certified to particular requirements laid down by the US National Institute of Science and Technology (NIST).

## Supplementary Information

Below is the link to the electronic supplementary material.

Supplementary file1 (DOCX 1837 kb)

Supplementary file2 (PDF 162 kb)

Supplementary file3 (XLSX 12 kb)
