# Supplementary material for: Quality assurance and quality control reporting in untargeted metabolic phenotyping: mQACC recommendations for analytical quality management
Source: Metabolomics. 2022 Aug 27;18(9):70. doi: 10.1007/s11306-022-01926-3 (PMC9420093; doi:10.1007/s11306-022-01926-3)
Supplement: Supplementary file 1 — Supplementary file1 (DOCX 1837 kb) [file 11306_2022_1926_MOESM1_ESM.docx]

**ONLINE RESOURCES**

**Quality Assurance and Quality Control Reporting in Untargeted Metabolic Phenotyping: mQACC Recommendations for Analytical Quality Management**

Jennifer A. Kirwan^1,2,3*^, Helen Gika^4*^, Richard D. Beger^5^, Dan Bearden^6^, Warwick B. Dunn^7^, Royston Goodacre^7^, Georgios Theodoridis^8^, Michael Witting^9^, Li-Rong Yu^5^, Ian D. Wilson^7,10*^ on behalf of the metabolomics Quality Assurance and Quality Control Consortium (mQACC)

^1^ Berlin Institute of Health at Charité – Universitätsmedizin Berlin, Metabolomics Platform, Anna-Louisa-Karsch-Str. 2, 10178 Berlin, Germany

2 Max Delbrück Center, Robert-Rössle Strasse 10, 13125 Germany

3 School of Veterinary Medicine and Science, University of Nottingham, Sutton Bonnington Campus, Loughborough, LE12 5RD

^4^ School of Medicine, Aristotle University of Thessaloniki, 54124 Thessaloniki, Greece, BIOMIC_Auth, Center for Interdisciplinary Research and Innovation (CIRI-AUTH), 57001 Thermi, Greece

^5^ Division of Systems Biology, U.S. Food and Drug Administration (FDA), National Center for Toxicological Research, Jefferson, AR 72079, USA

^6^ Metabolomics Partners, 1065 Fronie Drive, Nesbit, Mississippi, 38651, USA

^7^ Department of Biochemistry and Systems Biology, Institute of Systems, Molecular and Integrative Biology, University of Liverpool, BioSciences Building, Crown St., Liverpool, UK, L69 7ZB

^8^ Aristotle University of Thessaloniki, 54124 Thessaloniki, Greece, BIOMIC_Auth, Center for Interdisciplinary Research and Innovation (CIRI-AUTH), 57001 Thermi, Greece

^9^ Metabolomics and Proteomics Core, Helmholtz Zentrum München, Ingolstädter Landstraße 1, 85764 Neuherberg, Germany

^10^ Department of Metabolism, Digestion and Reproduction, Division of Systems Medicine, Imperial College London, Hammersmith Campus, London, W12 0NN, UK.

* Authors for correspondence [Jennifer.kirwan@bih-charite.de](mailto:Jennifer.kirwan@bih-charite.de), [gkikae@auth.gr](mailto:gkikae@auth.gr) and [i.wilson@imperial.ac.uk](mailto:i.wilson@imperial.ac.uk)

**Contents:**

**Table OR1:** On-line Resource Draft reporting template for QC sample reporting

**Table OR2.** The various types of QC samples used in untargeted metabolic profiling

**Table OR3.** Recommended QC metadata and rationale for reporting it in metabolomics studies

See also the Xcel spreadsheet version of Table OR1 which can be annotated as work progress on a project in order to keep track of the suggested data inclusion.

In addition, see the PDF **“Pro Forma Minimum reporting standards document”** which also provides a general guide to what should be provided in publications or submissions to data repositories.

**
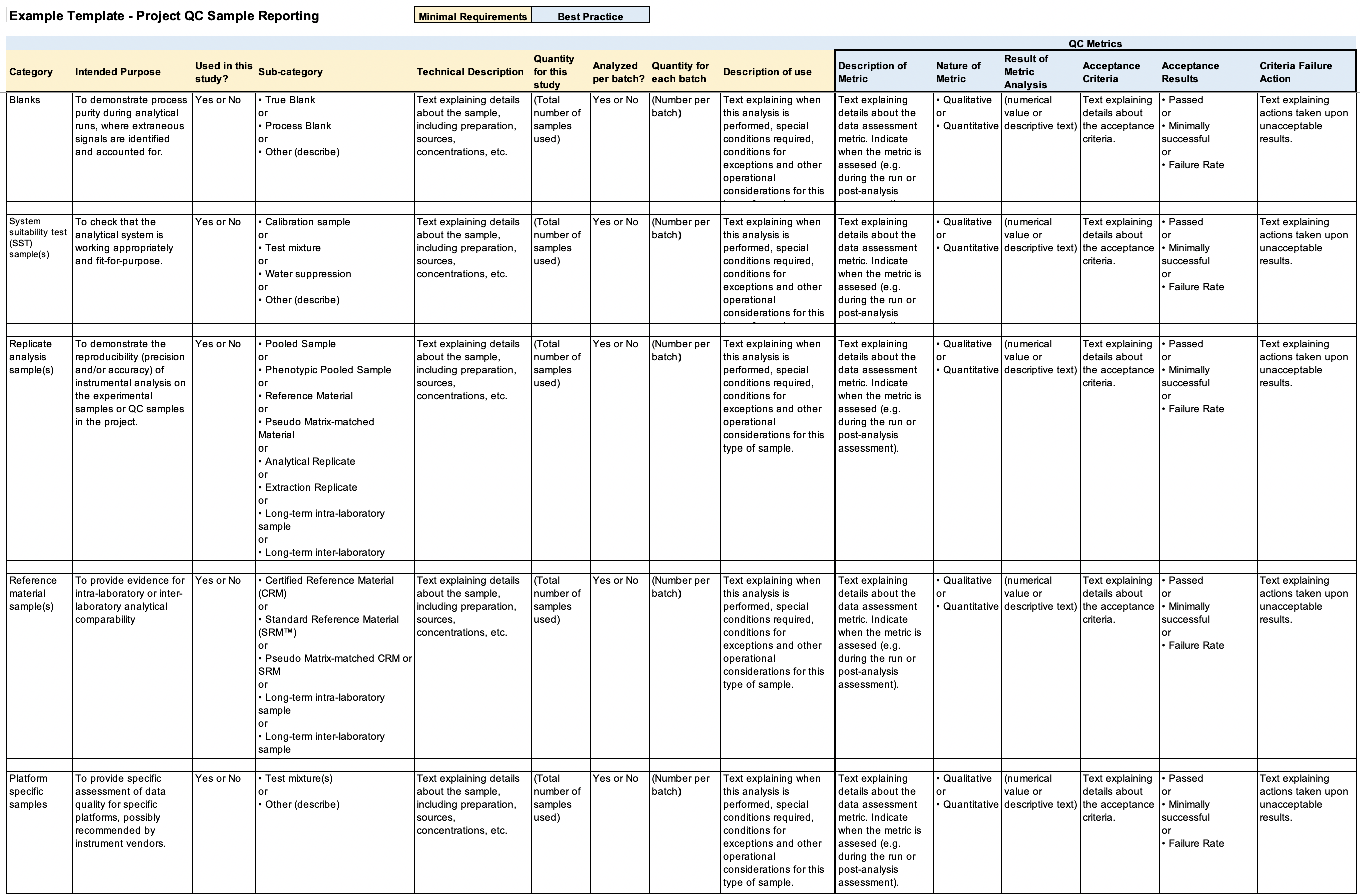
****Table OR1: On-line Resource Draft reporting template for QC sample reporting.**

**Table OR 2. The various types of QC samples used in untargeted metabolic profiling**

| **Type of QC Sample(s)** | **Alternative names** | **Purpose of QC Sample(s)** | **Considerations for QC sample composition** |
| --- | --- | --- | --- |
| System suitability QC samples |  | To demonstrate analytical system is “fit for purpose” and working within specification with no contamination, prior to analysis of study samples. | Should be consistent over long periods and potentially usable across multiple laboratories. A synthetic sample comprising a mixture of authentic chemical standards or reference material can be used for this purpose. |
| Intra-study QC samples | Within-study QC ‘pooled QC sample’  Intra-batch QC  Within-batch QC | To (a) condition the analytical system, (b) provide measures of within study reproducibility, (c) monitor, assess and potentially correct for systematic errors in measurements, e.g. drift in *m/z*, chemical shift, intensity and/or chromatographic retention time in QC samples (i.e. most often pooled samples) during a study and associated batches, to ensure that acceptance criteria are met for that specific study, and (d) optionally used to filter variables based on the linearity of their intensities in a dilution series of within-study QC. | Due to the nature of the algorithms applied to achieve these purposes, and because of matrix-specific effects on ionization, it is essential that the within-study QC is highly representative of the biological samples in the current study. It is practically derived from a small aliquot of all the individual biological samples within that study. These QCs are limited to one study. |
| Intra-lab QC samples | Within-lab QC ‘Long-Term Reference (LTR) QC’  Between-study QC | To assess (and potentially correct for) any differences between separate studies within one laboratory. Hence this type of QC has also been referred to as an ‘Inter- or between- study QC’. | Should be representative of the study samples and hence derived from a one-time pool of multiple extracted samples by a specific laboratory using a defined SOP, or a synthetic sample covering the relevant metabolite space or a reference material of sufficiently similar metabolic composition to the study samples. |
| Inter-lab QC samples | Between-lab QC (previously also known as a ‘Long-Term Reference (LTR) QC’) | To assess (and potentially correct for) any differences between individual laboratories. | Should be accessible to multiple laboratories, has known providence, is stable, characterised and available in controlled batch numbers. Ideally this QC has a similar metabolic composition or matrix to the biological samples in the study, although this is not always possible, in which case use as close to the same composition as possible. |
| Process blank samples | ‘blank’, solvent blank or ‘extraction blank’ | To enable the measurement of interfering signals (‘contaminants’) that may arise from the ‘process’ - e.g. from extraction solvents, plasticware, etc. - such that these contaminant signals can be removed from a study during the data processing. Sometimes used to assess carryover. | Study specific, prepared in the same manner as the biological samples except that no biological material is present. It is important to define the start and end points of the ‘process’ used to prepare this type of QC sample. |

**Table OR3. Recommended QC metadata and rationale for reporting it in metabolomics studies**

| **Metadata Category** | **Meta Data** | **Explanation** |
| --- | --- | --- |
| System suitability test (SST) | Test sample details | A full description of the sample(s) used for the SST should be given, similar in detail to that used for the QCs to enable a reader to assess the appropriateness and results of the test and reproduce it if required. |
|  | Test details | The full details of the test run(s), and the pass/fail criteria should give confidence to the reader that the instrument was reliably running. Pass/fail criteria should include aspects of instrument functionality that are specifically relevant to the analysis undertaken, e.g., negative ion mode results should be reported where negative ion mode was used for the analysis. |
| “Blank” samples | Sample preparation | Enables the reader to assess where contaminants in the blank may be arising from and how representative the blank is as a negative control. |
|  | Sample run order | Important to assess whether blank samples are measuring contamination or carryover. |
|  | Use in data analysis | Enables readers to assess research results and conclusions with knowledge about potential contamination or carryover that may influence results. |
| QC sample matrix details | Sample Preparation Date | Enables the reader to see how old the sample was and whether results may be influenced by chemical changes or degradation. |
|  | QC Matrix (cells, plasma, tissue etc.) | Matrix effects can have both positive and negative effects on metabolite intensities and will therefore influence results. |
|  | Storage Condition | Enables the reader to assess whether storage conditions were appropriate and whether results may be influenced by chemical changes or degradation. |
|  | Storage volume | Sublimation can occur in storage, even at low temperatures. The storage volume combined with the length of storage may contribute to sample variability. |
|  | Storage Temperature | Enables the reader to interpret whether storage conditions were appropriate and whether results may be influenced by chemical changes or degradation. |
|  | How were samples transported to lab | Enables the reader to interpret whether a cold chain was maintained, or whether transport conditions may influence results. |
| QC sample processing details | QC sample processing protocol including any extraction or dilution solvents used | Enables the reader both to assess and interpret results more completely (see text) and repeat them. |
|  | Number of Internal Standards (IS) (labeled or unlabeled) | Enables the reader both to assess and interpret results more completely (see text) and repeat them. |
|  | Chemical Identity of IS | Enables the reader both to assess and interpret results more completely (see text) and repeat them. In particular, the reader can critically analyse the appropriateness of any normalization or equivalent steps that have been performed with the internal standards. |
|  | Justification of commercial or homemade QC source | Both commercial and homemade QCs have advantages and disadvantages. How QC data can be used is dependent on what the QC sample is composed of. |
|  | Are QC samples measuring instrument variability or experimental variability | Were QC samples prepared just before samples are placed on the analytical instrument (and so measure only instrument variability) or have they undergone the identical extraction and processing procedures as the real samples (to measure the total of the experimental variability). Judging the performance of the QCs and the method in general is dependent on understanding what type of variability they are measuring. |
|  | Number of QC samples prepared | This gives important information about the number of QC samples prepared and analysed, which aids interpretation about their variability and whether some QC samples may have been excluded. |
| *Commercial QC* | Sample supplier | Enables a reader to repeat the experiment using the identical QC |
|  | Batch number | Enables a reader to identify possible causes of differences in an experimental outcome. |
|  | Number of freeze thaws | Enables interpretation due to freeze thaw effects. |
| *Homemade QC* | Preparation protocol | Enables the reader to both assess and repeat the experiment. |
|  | Which experimental samples were pooled to create the QC | Enables the reader to understand some of the results more properly, especially if data is made openly available. |
|  | How were pooled QCs stored throughout the study (aliquot size, dried down or not, temperature) | Enables the reader to interpret whether storage conditions were appropriate and whether results may be influenced by chemical changes or degradation. |
|  | Method of pooling (e.g., pre- or post- extraction) | Determines whether only instrument analytical variability, or total experimental variability is measured. |
| Analytical method | Confirm that the QCs were run as part of the same analytical batch as the samples discussed in the report | Confirms that the measured variability in the QCs represents the anticipated technical variability in the sample set. |
|  | Analytical method (if different from sample method) | Enables the reader to assess the method including e.g. the likelihood that chemical identities are probable given a stated method, and to reproduce the results. |
|  | Sample run order | Enables assessment and better understanding of intra-batch effects. |
|  | QC run details: frequency and intervals of QC samples run; randomized or block randomized | Enables assessment and better interpretation of results and subsequent processing e.g. some batch correction methods may be less reliable when performed using QCs which are not evenly distributed throughout the run, or where there are no “bookend QCs” i.e. a QC sample at the beginning and end of the run. This is also an opportunity for the writer to be clear about their QC handling procedure e.g. were any excluded from the final analysis and why. |
|  | Type of instrument and vendor | Enables the reader to assess the appropriateness of the experimental method and interpretation and to repeat the experiment if required. |
|  | Time elapsed since sample preparation (minimum and maximum) | Enables the reader to assess the likelihood of chemical degradation over the course of the run. |
| *Liquid/Gas chromatography* | Mobile phase (solvents or gas used) | Enables the reader to assess the appropriateness of the experimental method and interpretation and to repeat the experiment if required. May especially aid assessment of probability of chemical identifications. |
|  | Column used (manufacturer, chemistry, bead size, internal diameter, length) | Enables the reader to assess the appropriateness of the experimental method and interpretation and to repeat the experiment if required. May especially aid assessment of probability of chemical identifications. |
|  | Gradient | Enables the reader to assess the appropriateness of the experimental method and interpretation and to repeat the experiment if required. |
|  | Injection volume | Enables the reader to assess the appropriateness of the experimental method and interpretation and to repeat the experiment if required. |
|  | Split ratio (if used) | Enables the reader to assess the appropriateness of the experimental method and interpretation and to repeat the experiment if required. May also highlight experimental problems such as column overloading which may contribute to the final variability of the experiment. |
|  | Additional information (liners, vials used etc.) | Enables the reader to assess the appropriateness of the experimental method and interpretation and to repeat the experiment if required. Coloured vials may be particularly important for light sensitive chemicals for example, while the choice of liner used may affect the final detected intensities of certain compounds. |
| *Mass spectrometry* | MS method including resolution, *m*/*z* range, target ion selection parameters, collision energy | Enables the reader to assess the appropriateness of the experimental method and interpretation and to repeat the experiment if required. |
| *NMR* | Instrumental parameters including: 90-degree pulsewidth, pulse sequence name (or listing), spectral width, digitization info (number of datapoints), total recycle time, number of scans. | Enables the reader to assess the appropriateness of the experimental method and interpretation and to repeat the experiment if required. |
|  | Time for temperature stabilization | Enables accurate interpretation of results. |
| Reporting data | Report pass/fail criteria | Required to assess the appropriateness of the results. |
|  | Identification confidence | Enables assessment of the qualitative results and the biochemical interpretation. |
|  | Additional information | Includes information on how QC data was used to assess data reproducibility, batch drift and other information that improves the technical confidence of the research. |
